# Urban Ecological Environment Quality Evaluation and Territorial Spatial Planning Response: Application to Changsha, Central China

**DOI:** 10.3390/ijerph20043753

**Published:** 2023-02-20

**Authors:** Chan Lu, Lei Shi, Lihua Fu, Simian Liu, Jianqiao Li, Zhenchun Mo

**Affiliations:** 1 College of Architecture and Art, Central South University, Changsha 410075, China; 2 College of Urban and Environment, Hunan University of Technology, Zhuzhou 412007, China; 3 Hunan Provincial Key Laboratory of Safe Discharge and Resource Utilization of Urban Water, Zhuzhou 412007, China; 4 College of Geographic Sciences and Tourism, Hunan University of Arts and Science, Changde 415000, China; 5 College of Tourism, Hunan Normal University, Changsha 410081, China

**Keywords:** urban ecological environment quality, territorial spatial planning, RSEI, remote sensing, rapid urbanization, industrial land expansion, Changsha City

## Abstract

Scientific territorial spatial planning is of great significance in the realization of the sustainable development goals in China, especially in the context of China’s ecological civilization construction and territorial spatial planning. However, limited research has been carried out to understand the spatio-temporal change in EEQ and territorial spatial planning. In this study, Changsha County and six districts of Changsha City were selected as the research objects. Based on the remote sensing ecological index (RSEI) model, the spatio-temporal changes in the EEQ and spatial planning response in the study area during 2003–2018 were analyzed. The results reveal that (1) the EEQ of Changsha declined and then rose between 2003 and 2018, showing an overall decreasing trend. The average RSEI declined from 0.532 in 2003 to 0.500 in 2014 and then increased to 0.523 in 2018, with an overall decrease of 1.7%. (2) In terms of spatial pattern changes, the Xingma Group, the Airport Group and the Huangli Group in the east of the Xiangjiang River had the most serious EEQ degradation. The EEQ degradation of Changsha showed an expanding and polycentric decentralized grouping pattern. (3) Massive construction land expansion during rapid urbanization caused significant EEQ degradation in Changsha. Particularly, the areas with low EEQ were concentrated in the areas with concentrated industrial land. Scientific territorial spatial planning and strict control were conducive to regional EEQ improvement. (4) The prediction using the urban ecological model demonstrates that every 0.549 unit increase in NDVI or 0.2 unit decrease in NDBSI can improve the RSEI of the study area by 0.1 unit, thus improving EEQ. In the future territorial spatial planning and construction of Changsha, it is necessary to promote the transformation and upgrading of low-end industries into high-end manufacturing industries and control the scale of inefficient industrial land. The EEQ degradation caused by industrial land expansion needs to be noted. All of these findings can provide valuable information for relevant decision-makers to formulate ecological environment protection strategies and conduct future territorial spatial planning.

## 1. Introduction

With the rapid industrialization and urbanization of China, a large amount of peri-urban agricultural land has been transformed into urban construction land. Thus, the area of impervious urban surface has significantly expanded. Consequently, ecological land use with huge ecological value (e.g., vegetation and water bodies) has been gradually reduced and fragmented [1]. Urban construction land expansion has altered the function and structure of regional ecosystems [2,3]. In particular, in areas experiencing rapid urban expansion, the spatial patterns of the entire natural ecosystem are commonly damaged due to the intervention of human activities. Thus, this can lead to changes in natural ecological processes and biodiversity loss. Ecological security problems (such as soil erosion, forest vegetation degradation, air pollution and urban heat island effects) can threaten human health and sustainable economic and social development [4,5,6,7]. Globally, ecological environmental problems in China are severe. Currently, ecological civilization construction and high-quality development in China require changes in previous extensive and rapid urban spatial expansion, which has raised new urban planning and spatial control requirements. Therefore, the timely and accurate monitoring and assessment of the variations in urban ecological environment quality (EEQ) and the effect of rapid urbanization on EEQ are important for sustainable regional development.

Currently, many studies on ecological environment evaluation have been conducted on different scales, such as global [8], intercontinental [9], national [3,10], urban agglomeration [11], and cities [12]. Remote sensing, with its advantages of real-time, open, large-scale and rapid monitoring, has been extensively employed in the ecological environment field. It has developed into an effective method of dynamically monitoring elements of the ecological environment and evaluating changes in the regional ecological environment [13,14,15,16,17,18,19]. Using different remote sensing indices, many scholars have evaluated different ecosystems (e.g., cities [20,21], forests [22], grasslands [23], and watersheds [24]). These indices include the normalized difference impervious surface index (NDISI) for urban ecological environment evaluation [25], the normalized difference vegetation index (NDVI) for forest ecosystem change evaluation [26], the water body index for river information extraction and thus water environment evaluation [27], and land surface temperature (LST) for urban heat island effect evaluation [28]. These indices can better reveal the ecological characteristics of a specific aspect of ecosystems. However, the influencing factors of complex ecosystems are diverse. Thus, a single ecological index cannot comprehensively and accurately reflect the whole ecosystem and objectively evaluate ecological environment changes. It is necessary to obtain a comprehensive index in order to fully and objectively evaluate complex urban ecosystems (such as hills, urban areas, forestlands, farmlands, and wetlands).

The remote sensing ecological index (RSEI) was proposed by Xu [29] in 2013. This comprehensive ecological evaluation index integrates multiple ecological factors based on remote sensing information technology. RSEI integrates various factors (such as soil moisture, land surface temperature, vegetation cover and urban construction) to quantitatively and objectively reflect EEQ. It can also facilitate the visualization, temporal and spatial analysis, and trend prediction of regional EEQ variations. Thus, RSEI is more objective than other methods (e.g., the fuzzy synthetic evaluation method and the analytic hierarchy process). RSEI has been commonly utilized to evaluate regional EEQ in eastern coastal regions [11,12,30,31,32,33], western regions [34,35], urban agglomeration [36,37], new districts [38], watersheds [39,40,41,42], oases [43,44], and wetlands [45,46] in China. For example, based on the comprehensive nighttime light index and RSEI, Zheng et al. quantitatively evaluated the Chinese coastal zone during 2000–2019. Their findings presented increasing coupling coordination of coastal urbanization and EEQ. However, environmental improvement mainly occurred in non-urbanized areas. In urbanized areas, EEQ still faces severe problems [30]. Ji et al. examined the landscape pattern and EEQ changes in the Jing-Jin-Ji urban agglomeration during 2001–2015 using RSEI. They found that its landscape ecology exhibited the characteristics of aggregation, correlation, diversity and regularity, and anthropological activities had increasing effects on regional EEQ changes [11]. Hu et al. evaluated the EEQ of Fuzhou City in Fujian Province during 2000–2016 based on RSEI. They reported that the ecological environment in Fuzhou deteriorated significantly, and the RSEI values were distributed with low clusters at the urban center and high clusters at the edge [12]. Zhang et al. collected remote sensing images of Tianjin City in early summer, autumn, and winter in 1992, 2005, and 2018 and analyzed the seasonal and annual EEQ evolution of Tianjin using multitemporal analysis. They concluded that the EEQ of Tianjin generally improved, and the RSEI-based evaluation method was feasible [31]. In general, existing studies have mainly focused on coastal areas of China, while empirical studies on rapid urbanization areas in central China are relatively lacking. It is urgent to validate RSEI’s applicability using remote sensing data. Thus, this study provides data support for enriching the ecological environment evaluation methods that are suitable for rapid urbanization areas in central China. In addition, most existing studies on coastal cities have only analyzed the EEQ of ecosystems and their changes. Studies on the intrinsic mechanisms influencing ecological environment changes are insufficient, and there are fewer studies from the perspective of territorial spatial planning. However, scientific territorial spatial planning is of great significance to the improvement of urban EEQ. Therefore, it is crucial to evaluate the interrelationship of ecological environment changes with territorial spatial planning in depth. This can facilitate formulating practical ecological environment protection and control measures during territorial spatial planning and promote sustainable regional EEQ development.

As a new economic growth sector, the Chang-Zhu-Tan metropolitan area is a valuable platform for the rise of central China. Changsha City is the core of the Chang-Zhu-Tan metropolitan area and the central city of the urban agglomeration in the middle reach of the Yangtze River. Changsha has a typical hilly landscape with low ecological stability and high ecological sensitivity and belongs to a typical fragile ecosystem [47]. From 2003 to 2018, the urban expansion of Changsha accelerated: the population increased from 6,017,600 to 8,154,700; the urbanization rate increased from 49.16% to 79.12%; and the total GDP exceeded a trillion. Rapid urbanization and industrialization have transformed vegetation, wetlands, and agricultural land into construction land, causing various ecological environment problems such as urban heat islands, forest fragmentation, a sharp reduction in arable land, and land degradation [11]. It is urgent to systematically evaluate the EEQ of Changsha. Changsha County and six urban districts of Changsha City were taken as the study area in this work. Based on Landsat remote sensing datasets, the temporal and spatial EEQ changes in Changsha and its influencing factors due to rapid urbanization during 2003–2018 were analyzed using the RSEI model. This study had the following objectives: (1) to quantitatively analyze the EEQ of Changsha City during 2003–2018 using RSEI and objectively evaluate its spatial and temporal changes; (2) to determine the areas with degraded, unchanged, and improved EEQ in Changsha; (3) to analyze the intrinsic mechanisms affecting EEQ changes, including the influence of urbanization, industrial land expansion, and territorial spatial planning; and (4) to simulate and predict the trend of EEQ changes in Changsha using the urban ecological model. The study findings can provide a decision-making reference and scientific analysis for future territorial spatial control and planning.

## 2. Materials and Methods

### 2.1. Description of the Study Area

**Figure 1:**
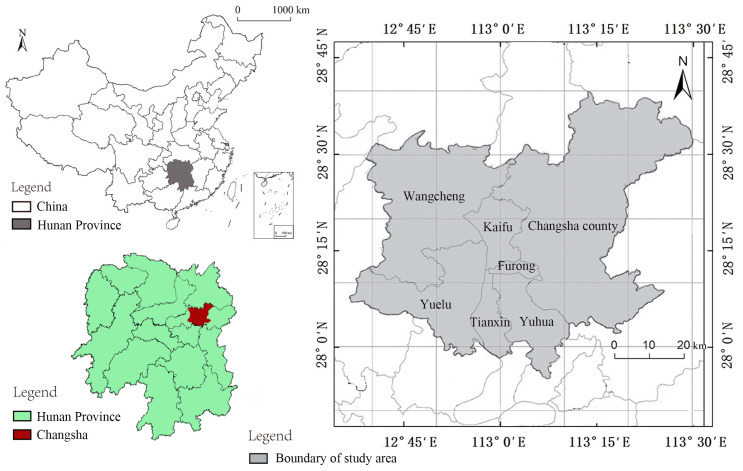
Location of the study area.

As the capital city of Hunan Province, Changsha City also serves as a pilot area for the national “two-type society” comprehensive support reform, and is an important node city in the middle reaches of the Yangtze River urban agglomeration and economic belt and the core of the Chang-Zhu-Tan metropolitan area. In this study, Furong District, Kaifu District, Tianxin District, Yuhua District, Yuelu District, Wangcheng District, and Changsha County of Changsha City were selected as the research objects, with a total area of 3952.45 km^2^, as shown in Figure 1. The usual resident population of the six districts and Changsha County was 5,498,400 at the end of 2018. The scale and total amount of regional economic development ranked 1st in Hunan Province. The climate of Changsha is a typical subtropical monsoon climate (with cold winters and hot summers). The annual average temperature and the total annual precipitation are 18.5 °C and approximately 1318.3 mm, respectively. The terrain is undulating, with various landforms and a well-developed surface water system. The Xiangjiang River runs through the city. Alluvial plains with low terrain have developed on both banks of the Xiangjiang River. There are low mountains and hills with high terrain at the east, west, and southeast sides.

### 2.2. Data Source and Processing

The land use and cover change (LUCC) datasets were attained from the Resource and Environmental Science Data Center (RESDC) at the Chinese Academy of Sciences (https://www.resdc.cn) (accessed on 1 October 2021). The satellite remote sensing imagery data were downloaded from the official website of the United States Geological Survey (USGS) (https://www.usgs.gov) (accessed on 20 October 2021), including the remote sensing images of 17 January 2003 (Landsat 7 ETM+), 8 December 2008 (Landsat 5 TM), 23 January 2014, and 3 February 2018 (Landsat 8 OLI). These four years were chosen to be consistent with the formulation and revision time of the Changsha City Master Plan (2003–2020). These four datasets have a resolution of 30 m. The cloud cover of the four images that were chosen was less than 10% in order to lessen the effect of cloud cover on data quality. These images have similar seasonal phases, thus facilitating comparison. The four datasets were pre-processed using ENVI 5.5 for atmospheric correction, radiometric calibration, seamless mosaic, geometric correction, and image cropping. The quadratic polynomial function was used to achieve geometric correction. The nearest neighbor pixel method was used for different images, with the root mean square error less than 0.5 pixels. Since large-scale water bodies affect the RSEI accuracy, surface water bodies, such as the Xiangjiang River, the Liuyang River, the Meixi Lake, and the Songya Lake were masked.

### 2.3. Methods

#### 2.3.1. RSEI Calculation

**Figure 2:**
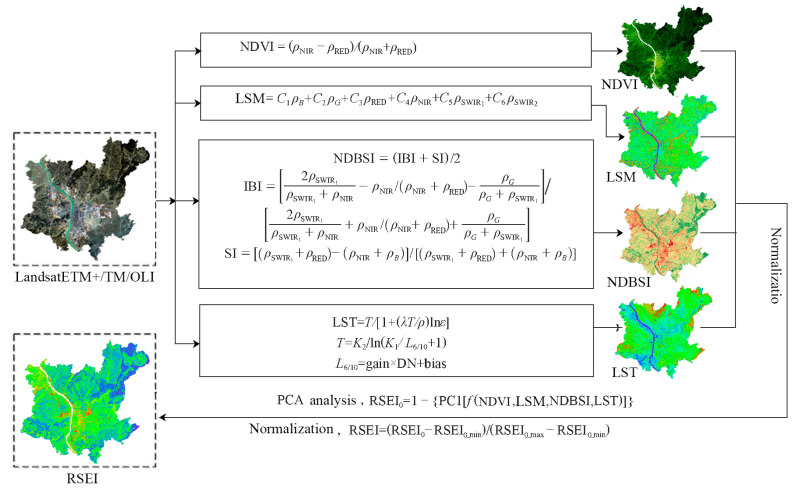
Flowchart of RSEI calculation.

RSEI includes four component indicators, i.e., land surface moisture (LSM), normalized difference vegetation index (NDVI), normalized difference building–soil index (NDBSI) and land surface temperature (LST) [10]. These indicators showed significant correlations with ecological conditions. Therefore, they are commonly used to assess ecosystems. The flowchart for calculating RSEI is presented in Figure 2. The method of calculating each ecological indicator is as follows:

(1) Normalized difference vegetation index

NDVI represents greenness and is significantly correlated with leaf area index, plant biomass, and vegetation cover [48]. NDVI is expressed as

(1) $$\mathrm{NDVI}=\left(\rho_{nir}-\rho_{red}\right)/\left(\rho_{nir}+\rho_{red}\right)$$

where ρ denotes the spectral reflectance of the corresponding waveband.

(2) Land surface moisture

LSM represents wetness and indicates soil moisture content, vegetation, and water bodies [12]. LSM is significantly correlated with EEQ. Low LSM values indicate low vegetation cover, severe land degradation, and a poor ecological environment; high LSM values indicate rich land vegetation cover, sufficient soil moisture, and a good ecological environment. The sensors for Landsat-7 ETM+, Landsat-5 TM, and Landsat-8 OLI imagery are different. Thus, extraction equations of their moisture indicators [49,50] vary:

(2) $$\begin{array}{c} {\mathrm{LSM}}_{\mathrm{ETM}+}=0.2626\rho_{blue}+0.2141\rho_{green}+0.0926\rho_{red}+0.0656\rho_{nir}\\ -0.7629\rho_{swir1}-0.5388\rho_{swir2} \end{array}$$

(3) $$\begin{array}{c} {\mathrm{LSM}}_{\mathrm{TM}}=0.0315\rho_{blue}+0.2021\rho_{green}+0.3102\rho_{red}+0.1594\rho_{nir}\\ -0.6806\rho_{swir1}-0.6109\rho_{swir2} \end{array}$$

(4) $$\begin{array}{c} {\mathrm{LSM}}_{\mathrm{OLI}}=0.1511\rho_{blue}+0.1973\rho_{green}+0.3283\rho_{red}+0.3407\rho_{nir}\\ -0.7117\rho_{swir1}-0.4559\rho_{swir2} \end{array}$$

where ρ indicates the reflectance for each waveband of ETM+, TM, and OLI images.

(3) Normalized difference building–soil index

NDBSI represents dryness and is the average of the soil index (SI) and the building index (IBI) [40,51].

Human activities strongly influence ecological conditions. Adversely, natural ecological land is converted into impervious construction land, leading to surface drying. NDBSI is used to indicate the surface dryness in the area and is expressed as

(5) NDBSI=(SI+IBI)/2

(6) $$\mathrm{SI}=\left[\left(\rho_{swir1}+\rho_{red}\right)-\left(\rho_{nir}+\rho_{blue}\right)\right]/\left[\left(\rho_{swir1}+\rho_{red}\right)+\left(\rho_{nir}+\rho_{blue}\right)\right]$$

(7) $$\begin{array}{l} \mathrm{IBI}=\{2\rho_{swir1}/\left(\rho_{swir1}+\rho_{nir}\right)\\ \;-\left[\rho_{nir}/\left(\rho_{nir}+\rho_{red}\right)+\rho_{green}/\left(\rho_{green}+\rho_{swir1}\right)\right]\}\\ \;/\{2\rho_{swir1}/\left(\rho_{swir1}+\rho_{nir}\right)\\ \;+\left[\rho_{nir}/\left(\rho_{nir}+\rho_{red}\right)+\rho_{green}/\left(\rho_{green}+\rho_{swir1}\right)\right]\} \end{array}$$

(4) Land surface temperature

LST represents the thermal component and is significantly correlated with surface water circulation, vegetation growth, and urbanization. It can be employed as a thermal indicator to respond to the land surface ecological environment. In this study, based on inversion using the atmospheric correction method, LST is calculated [52]:

(8) L=gain×DN+bias

(9) $$T=K_{2}/\ln\left(K_{1}/L+1\right)$$

(10) LST=T/[1+(λT/ρ)lnε]

where DN is the grayscale value of each pixel; L is the radiation value in thermal infrared bands; bias and gain are band bias and gain, respectively, which can be obtained from image header files; $K_{1}$ and $K_{2}$ are calibration parameters and can be obtained from user manuals; T is the temperature at the sensor; λ is the central wavelength of thermal infrared bands, i.e., 11.5 μm for the sixth band of TM data, 11.457 μm for the sixth band of ETM+ data, and 10.9 μm for the tenth band of OLI data; ε is the surface specific emissivity; and ρ = 1.438 × 10^−2^ m∙K. 

#### 2.3.2. RSEI Evaluation Model

The above four component indicators are integrated using principal component analysis (PCA) to automatically quantify the contribution of each index to ecology. The four component indicators need to be normalized before the RSEI calculation for unified dimensions between [0, 1]. The initial RSEI (RSEI_0_) is calculated based on the contribution of the component indicator to the first principal component (PC1). Then, RSEI_0_ is normalized to construct the final RSEI [53]. $RSEI_{0}$ and RSEI are expressed as

(11) $$RSEI_{0}=1-\left\{PC_{1}\left[f\left(NDVI,\;LSM,\;LST,\;NDBSI\right)\right]\right\}$$

(12) $$RSEI=\left(RSEI_{0}-RSEI_{0\_min}\right)/\left(RSEI_{0\_max}-RSEI_{0\_min}\right)$$

RSEI values vary between [0, 1]. An RSEI value closer to 1 indicates better EEQ.

## 3. Results and Analysis

### 3.1. Principal Component Analysis Results of RSEI

Table 1 presents the PCA results of Changsha in 2003, 2008, 2014, and 2018. (1) The contribution rates to PC1 eigenvalues exceeded 75%. This indicates that PC1 contained the most information of the component indicators (NDVI, LSM, NDBSI, and LST). These four indicators can be reasonably used to construct the RSEI model of the study area and analyze the temporal and spatial changes of Changsha’s EEQ. (2) According to the contribution analysis of the component indicators, LST and NDBSI were negative, while LSM and NDVI were positive. This was in agreement with the fact that wetness and greenness positively affect EEQ, while temperature and dryness have negative effects [54]. (3) Further comparison of the absolute PC1 loading values reveals that the absolute contribution rate of NDBSI to PC1 was the largest. This indicates that the impervious surface (i.e., construction land) had the largest influence on the EEQ evaluation results in Changsha City. Urban construction land expansion is the most important factor affecting the EEQ changes in Changsha.

### 3.2. Analysis of Spatial-Temporal Changes in the EEQ of Changsha

Table 2 presents the average values of LSM, NDVI, NDBSI, LST, and RSEI. In the study area, during 2003–2018, RSEI decreased and then increased. EEQ generally exhibited an overall downward trend. RSEI decreased from 0.532 in 2003 to 0.500 in 2014 (by 6.02%) and increased from 0.500 in 2014 to 0.523 in 2018 (by 4.6%). The RSEI value showed an overall decrease of 1.69%. 

**Figure 3:**
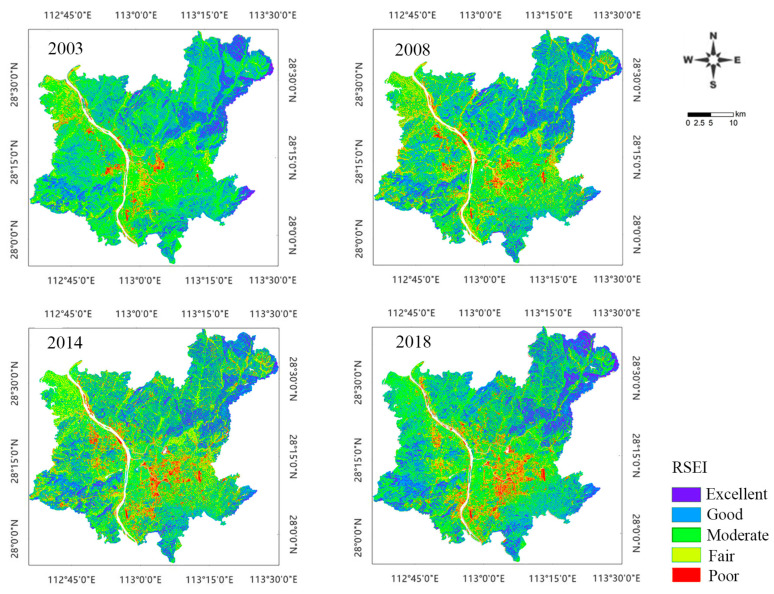
Spatio-temporal distribution of different RSEI levels in Changsha in 2003, 2008, 2014, and 2018.

To further explore the EEQ changes in the study area, the RSEI values for each year were divided into five levels at an interval of 0.2. Figure 3 provides the spatial and temporal EEQ distribution in Changsha during 2003–2018. Red, yellow, green, light blue, and dark blue represent poor, fair, moderate, good, and excellent EEQ levels, respectively. The spatial distribution pattern (Figure 3) shows that the areas with poor EEQ in 2003 were small and scattered, and were relatively concentrated in the Xingsha high-tech development zone and Hexi economic and technological development zone. The areas with excellent EEQ were mainly the periphery of the main urban area, such as Yujingping Town in the southern part of Yuelu and the towns of Shuangjiang, Jinjing, Gaoqiao, and Lukou in the northern part of Changsha County. These areas are mostly low mountains and hills with high terrain, high vegetation coverage and good EEQ. Compared with that in 2003, the red areas with poor EEQ in 2008, 2014, and 2018 gradually expanded, showing the characteristics of polycentric grouping expansion. Particularly, the areas with low EEQ values converged in the southeast direction. The areas in the southeast direction include the Xingma Group, the Airport Group, and the Huangli Group, which are mostly industrial parks with serious ecological degradation. The Muyun Group and the Pingpu Group in the south have rapidly grown in terms of construction land due to the southward urban expansion of integrated Chang-Zhu-Tan development. The ecological environment in these areas has started to deteriorate.

Table 3 provides the area and percentage at each RSEI level in 2003, 2008, 2014, and 2018. The EEQ of Changsha declined and then increased during 2003–2018, with an overall decreasing trend. Specifically, the areas with moderate and good EEQ levels decreased from 70.81% in 2003 to 52.4% in 2014 and then increased to 62.84% in 2018. The areas with fair/poor EEQ levels increased from 22.41% to 37.73% during 2003–2014 and then decreased to 27.19% in 2018. The percentage of the areas with excellent EEQ improved from 6.78% to 9.97%. This indicates that although the ecological environment was degraded due to construction land expansion, the ecological environment management measures in Changsha have significantly improved EEQ in local areas. This is in line with the urban development situation in Changsha in recent years. In December 2007, the Chang-Zhu-Tan urban agglomeration was approved by the State Council as a pilot zone for national environmentally friendly and resource-saving society construction comprehensive support reform. In order to implement the construction requirements of a two-type society and an ecologically livable city, the Changsha municipal government conducted pollution treatment and ecological restoration in key areas. The Changsha City Master Plan (2003–2020) required strengthening the protection and control of wetland resources and building large parks and ecological green areas. Thus, the local urban environment was significantly improved. Although urbanization can degrade the ecological environment, strict protection and control measures can maintain or even improve it.

### 3.3. Analysis of Spatial and Temporal Differences in the EEQ of Changsha

**Figure 4:**
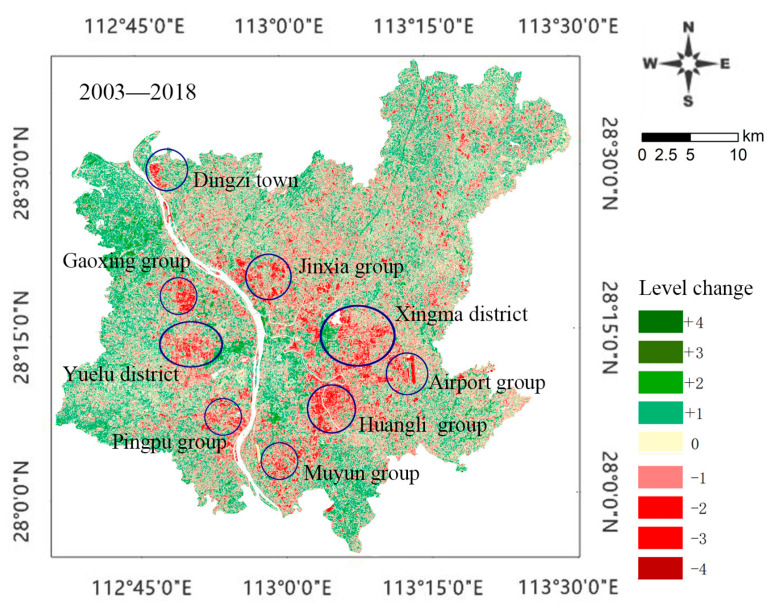
Changes of the RSEI during 2003–2018.

We analyzed spatial and temporal differences in the EEQ of Changsha from 2003 to 2018 based on RSEI values (Figure 4). The green indicates the areas with improvement of the EEQ, and the darker green indicates more significant EEQ improvement. The red indicates the areas with degradation of the EEQ, and the darker red indicates more serious ecological degradation. The areas with insignificant EEQ changes are shown in yellow. From Figure 4, it can be seen that the dark green areas were mainly concentrated in the central urban areas, along rivers, around lakes and in peripheral towns, with significantly improved EEQ. The dark red areas were mainly concentrated in the Xingma Group, the Airport Group, and the Huangli Group in the southeast, with seriously degraded EEQ. Several groups (such as Jinxia, Muyun, Gaoxing, and Pingtang) also showed degraded EEQ. The EEQ degradation in Changsha showed a polycentric and decentralized grouping pattern. Table 4 shows the statistical results of the changes in RSEI levels in Changsha during 2003–2018. The EEQ changes can be divided into three categories, i.e., degraded, unchanged, and improved. The degraded and improved categories can be further divided into four levels, expressed as ±4, ±3, ±2, and ±1 according to the significance. The statistical results indicate that during 2003–2018, the areas with unchanged, improved, and degraded EEQ accounted for 46.15% (1759.21 km^2^), 26.91% (1025.74 km^2^), and 26.94% (1027.27 km^2^) of the total area, respectively.

## 4. Discussion

### 4.1. Impact of Urbanization Process on the EEQ of Changsha

**Figure 5:**
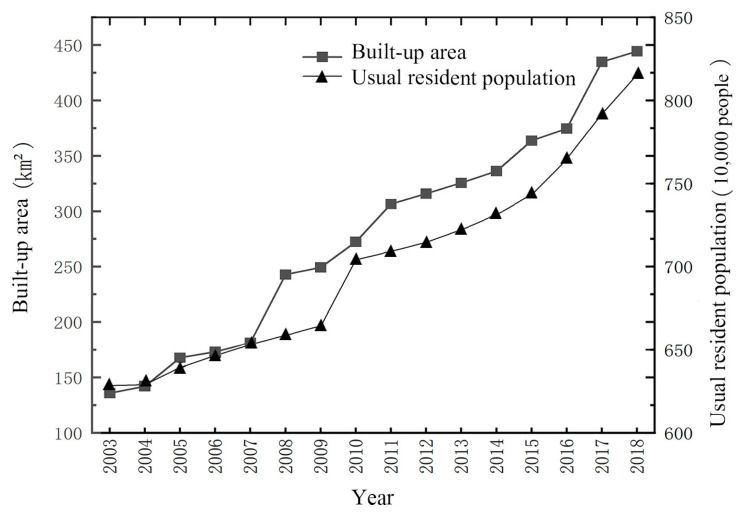
Changes in built-up areas and usual resident population in Changsha during 2003–2018.

**Figure 6:**
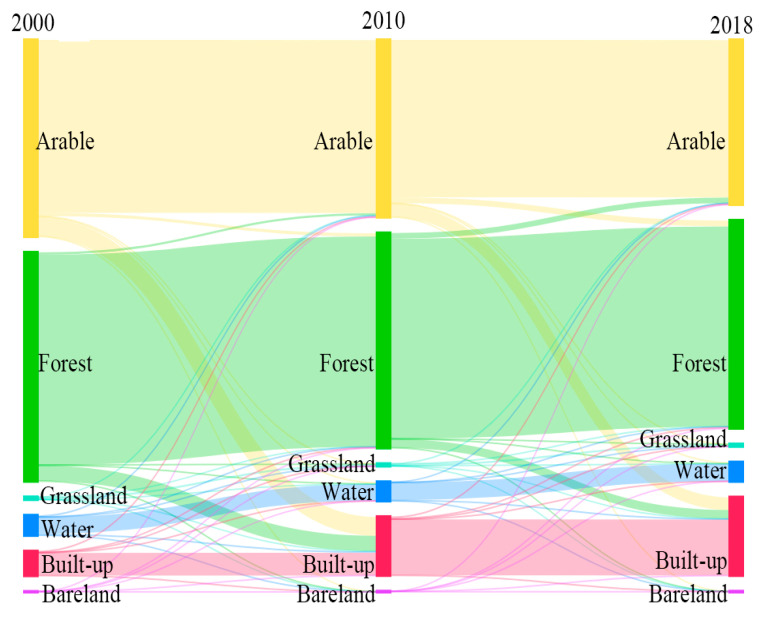
Sankey diagram of land use/cover types in Changsha during 2003–2018.

During the interaction between urbanization and EEQ, high-intensity urbanization will inevitably disturb or even damage the local ecological environment. The ecological environment deterioration will, in turn, restrict urbanization and sustainable development [55]. From 2003 to 2018, the urbanization rate of Changsha increased from 49.16% to 79.12%. The usual resident population of Changsha increased from 6,283,400 in 2003 to 8,154,700 in 2018. The built-up area rose sharply from 135.84 to 444.36 km^2^ (Figure 5). The rapid urbanization development led to a siphon effect, thus prompting accelerated urban spatial expansion and significant land use conversion [56]. Land use changes can cause a shift in regional mass and energy cycling, thus significantly affecting the regional ecological environment [57]. The Sankey diagram of land use changes in Changsha (Figure 6) shows that from 2003 to 2018, construction land areas in Changsha showed the largest increase among all land use types, mainly attributed to the conversion of arable land and forestland. This indicates that Changsha experienced significant construction land expansion during 2003–2018. Combined with Table 2, the rapid urbanization in Changsha during 2003–2014 increased the urban impervious surface area and land surface temperature while reducing urban vegetation and green space. Thus, these changes led to ecological degradation and affected regional sustainable development.

**Figure 7:**
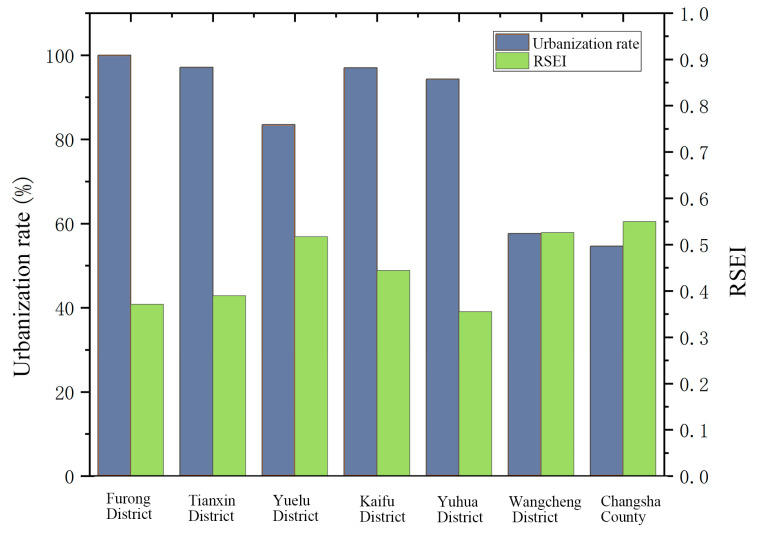
Comparison of RSEI and urbanization rates of each district in Changsha in 2018.

To examine the relationship between urbanization and RSEI, the RSEI values and urbanization rates of seven administrative districts in Changsha City in 2018 were analyzed (Figure 7). Based on the correlation analysis using SPSS, RSEI showed a highly significant negative correlation with the urbanization rate (R = −0.859, *p* = 0.013 < 0.05). This indicates that during the study period, urbanization negatively affected the EEQ improvement. In particular, the degradation of regional EEQ was mainly attributed to the increased area of impervious surfaces due to construction land expansion. These findings are consistent with previous studies of Nanjing city [58]. Traditional rapid urbanization only focuses on the urban scale and population size instead of development quality and EEQ. It is urgent to improve regional urban EEQ through new urbanization.

### 4.2. Impact of Industrial Land Expansion on the EEQ of Changsha

**Figure 8:**
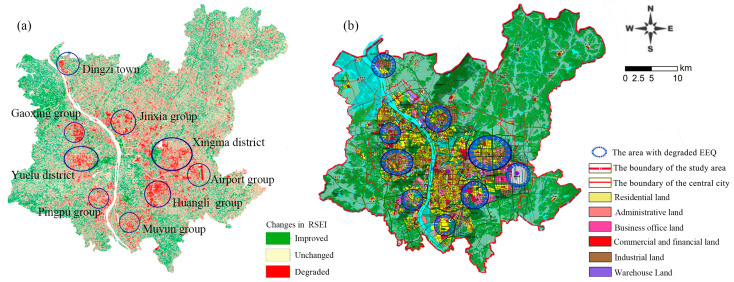
RSEI changes and land use planning: (**a**) RSEI changes during 2003–2018; (**b**) land use planning coordination map of the Changsha City master plan (2003–2020) (2014 revised version). Source: Changsha City planning information service center.

**Figure 9:**
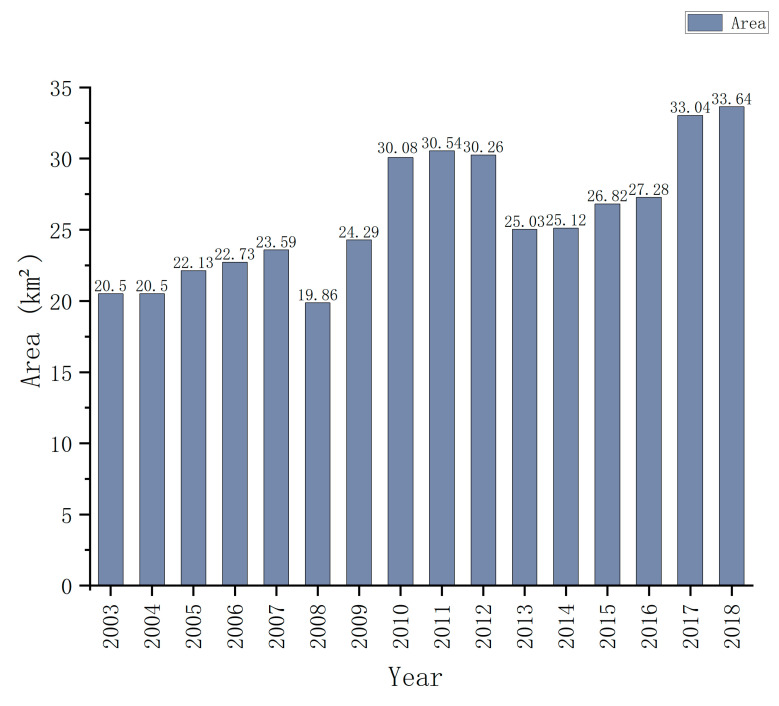
Statistics of industrial land area in Changsha from 2003 to 2018. Data sources: China city construction statistical yearbook from 2003 to 2018 (https://www.mohurd.gov.cn) (accessed on 28 October 2021).

**Figure 10:**
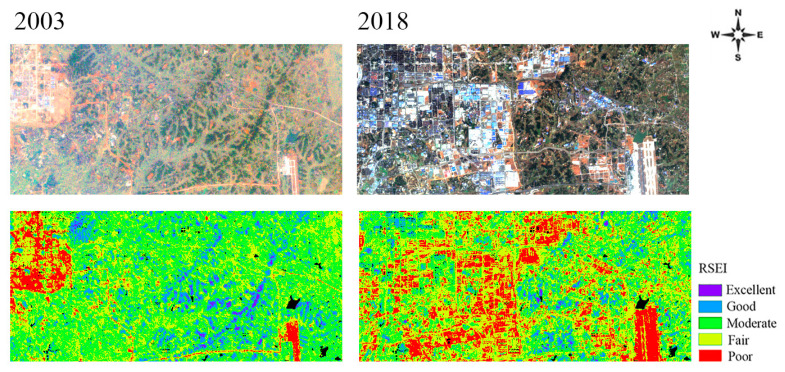
Original and corresponding RSEI images of Xingma District in Changsha in 2003 and 2018.

Massive industrial land expansion is the key factor causing EEQ degradation in Changsha. The areas with concentrated industrial land have exhibited the most serious EEQ degradation. These findings are different from previous studies in Tianjin city [31]. As can be seen in Figure 8, the areas (in red) with degraded EEQ were mostly the areas with concentrated industrial land in Changsha City Master Plan (2003–2020). In 2005, Changsha introduced new industrialization development planning: following an industrial development path of parkization, clustering, and intensification and relocating main urban industries out of the city. Thus, industrial land expanded rapidly. The annual statistical data of the industrial land area in Changsha City (Figure 9) shows that the industrial land area of Changsha City increased by 13.14 km^2^ from 2003 to 2018, an increase of 64.1%. Industrial clusters, including the national high-tech industrial development zone in the Yuelu District, the Xingma Group, the Airport Group, the Jinxia Group, and the Gaoxing Group, have contributed more than 70% of the gross output of industries above the designated size in Changsha. These areas are mostly industrial bases for high-tech, machinery manufacturing, and aviation industries, as well as logistics centers. Built-up industrial land generally has a high building density and a low greening rate. The natural ground surface is greatly reduced; thus, surface transpiration and evapotranspiration are seriously inhibited. Consequently, this results in the most serious EEQ degradation in industrial land. For example, in a typical industrial land use (i.e., the Xingma Group), the satellite and RSEI images in 2003 and 2018 (Figure 10) show that this area was generally covered with industrial plants in 2018, and a large amount of the original forestland and farmland in the area was damaged in 2003. The original blue area representing good EEQ was replaced by the red area representing poor EEQ. The increased impervious surface coverage of buildings and decreased green space led to a sharp decline in the regional EEQ. In the future, strengthening and reasonably controlling intensive and efficient industrial land use and preventing further EEQ deterioration are important measures to enhance the EEQ of Changsha and realize sustainable and healthy regional development.

### 4.3. Impact of Territorial Spatial Planning on the EEQ of Changsha

**Figure 11:**
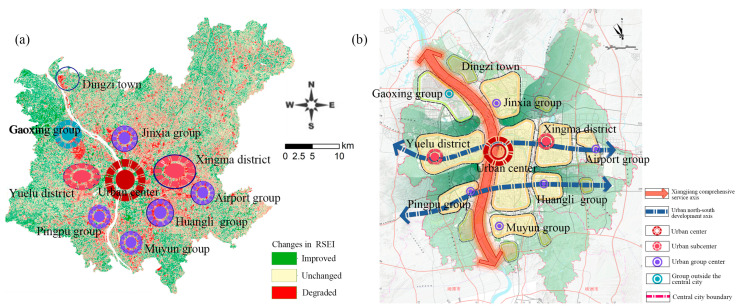
Spatial structure: (**a**) spatial distribution structure of RSEI level changes in Changsha during 2003–2018; (**b**) central city spatial structure planning map in Changsha City master plan (2003–2020) (2014 revised version). Source: Changsha City planning information service center.

Territorial spatial planning positively affects the changes in urban functional layout and EEQ in Changsha. Figure 11 shows that the EEQ improvement had a lumpy and leap spatial distribution, mainly in the central urban area, along rivers, and around various lakes and peripheral towns. The EEQ improvement in these areas was attributed to the 2014 revision of the Changsha City Master Plan (2003–2020). This planning involved transforming old areas in central urban areas, actively conducting reforestation, increasing wetland park planning, building large ecological green spaces and forest parks, and strengthening the planning control of nature reserves and scenic spots. Changsha has made every effort to promote the comprehensive management and ecological restoration of all rivers, effectively improving EEQ in these areas. The newly built Shawan Park, Xiangfu Cultural Park, Jianshan Lake Park, and Gushan Sports Park have also optimized the urban ecological pattern. Due to strict ecological control, the ecological environment of important ecological nodes and barrier areas, such as the Yuelu Mountain Scenic Spot, has been improved. Tiaoma Town on the periphery of the main urban area has gradually improved EEQ and become an important ecological barrier for the Chang-Zhu-Tan urban agglomeration after scientific planning and strict protection due to its inclusion in the Chang-Zhu-Tan Green Heart Area. Figure 11a shows that the area with EEQ degradation exhibited a pattern of expanding to the southeast and polycentric decentralized grouping. Figure 11b shows the urban spatial structure of Changsha, with “one center, two subcenters and five groups”, as established in the Changsha City Master Plan (2003–2020) (2014 revised version). A comparison of Figure 11a,b indicates that the spatial distribution structure of the RSEI level changes was consistent with the structure of Central City Spatial Structure Planning. As a top-level strategic plan, the territorial spatial planning determines the spatial pattern changes of urban EEQ. Scientific territorial spatial planning and strict control can facilitate EEQ improvement, which is consistent with the published literature of Pingtan Comprehensive Experimental Zone in Fuzhou city [32].

### 4.4. EEQ Development Trend of Changsha

To further analyze the EEQ status of Changsha quantitatively, an urban ecological model was developed in order to predict the changing trend of urban EEQ. First, 1176 sample points were randomly collected from RSEI images of the study area in 2018 (excluding some invalid points of water bodies). Then, a stepwise regression analysis was conducted. RSEI was used as the dependent variable, and LSM, NDVI, NDBSI, and LST were used as the independent variables. Finally, an urban ecological model was developed based on RSEI and passed the significance test at 1%:

RSEI = 0.232LSM + 0.182NDVI − 0.494NDBSI − 0.108LST + 0.607 (R^2^ = 0.963)

The coefficients of each indicator variable show that the absolute value of NDBSI was significantly higher than other variables. This further verifies that urban construction land expansion during urbanization had the most significant impact on the EEQ changes in Changsha.

**Figure 12:**
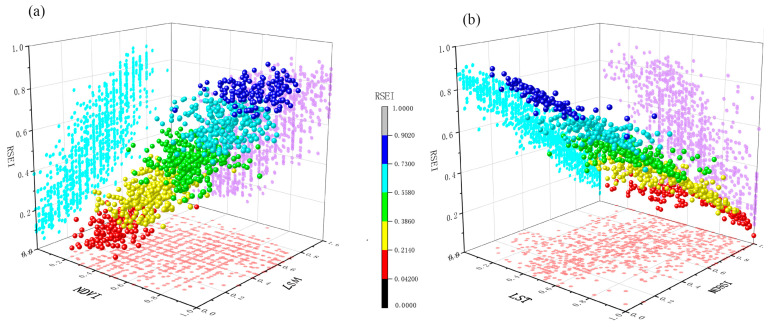
Three-dimensional scatter plots of the sampling points: 3D space for (**a**) LSM, NDVI, and RSEI; (**b**) LST, NDBSI, and RSEI.

The indicators were projected into three-dimensional space to test the relationship of RSEI with each indicator (Figure 12). The results show that LSM and NDVI positively correlated with EEQ, and LST and NDBSI negatively correlated with EEQ.

The model prediction shows that every 0.549 unit increase in NDVI or 0.2 unit decrease in NDBSI can improve the RSEI of the study area by 0.1 unit, thus improving EEQ. Therefore, increasing green space while reducing equivalent building land will achieve twice the result with half the effort. In the future urban planning and construction of Changsha, the increased proportion of impervious surfaces (particularly inefficient industrial land) should be moderately controlled. In addition, the construction and investment of forest parks and urban green space need to be increased to create an ecologically sound and sustainable urban living space.

### 4.5. Strengths and Limitations

This research can serve as a theoretical basis for objectively evaluating the EEQ of Changsha City. Based on the RSEI model, dynamic historical monitoring and the spatio-temporal change analysis of EEQ in Changsha were conducted. The EEQ level was spatially visualized rapidly and objectively by extracting corresponding indicators from remote sensing images. In addition, non-natural factors affecting regional EEQ changes, such as the urbanization process, industrial land expansion, and territorial spatial planning, were discussed.

Although the RSEI model has advantages for quantitatively conducting EEQ evaluation, it still has some limitations. The evaluation method mainly focuses on four component indicators (i.e., wetness, greenness, dryness, and heat). However, the factors affecting EEQ are complex and diverse. These four indicators are insufficient to comprehensively assess changes in ecosystems and environments. Future research is needed to improve the model using more diverse spatial data, such as land resources, terrain topography, and air quality data. In addition, Landsat 5 TM/Landsat 7 ETM+/ Landsat 8 OLI remote sensing images were used in our study. The uncertainty caused by the Landsat images from different sensors could affect the evaluation results. In order to obtain more comprehensive results, we also intend to use multi-temporal and multi-seasonal data for comparison and analysis.

Overall, even though some limitations exist, this study is of practical significance in improving regional EEQ. It can provide references for policymakers of territorial spatial planning and ecological environment protection to develop more specific EEQ improvement measures for the next stage of territorial spatial planning.

## 5. Conclusions

This study quantitatively evaluated the temporal and spatial changes in the EEQ of Changsha City during 2003–2018 using the RSEI model. The findings show that the EEQ of Changsha experienced fluctuating changes in this stage, first decreasing and then increasing slightly. EEQ generally exhibited an overall downward trend. The area with fair and poor EEQ increased from 22.41% in 2003 to 27.19% in 2018. The areas with good and moderate EEQ decreased from 70.81% in 2003 to 62.84% in 2018. The areas with unchanged, improved, and degraded EEQ from 2003 to 2018 accounted for 46.15%, 26.91%, and 26.94% of the total area, respectively. Spatially, EEQ degradation mainly occurred in Xingma area, the Airport Group, and the Huangli Group in the southeast and showed a polycentric decentralized grouping pattern. The areas with improved EEQ were mostly located in the central urban area, along the rivers, around the lakes, and in peripheral towns. Massive construction land expansion during the process of rapid urbanization caused the significant EEQ degradation of Changsha. Particularly, areas with low EEQ converged in the areas with concentrated industrial land. Scientific territorial spatial planning and strict control are conducive to regional EEQ improvement. The prediction of the proposed urban ecological model shows that every 0.549 unit increase in NDVI or 0.2 unit decrease in NDBSI in the future would improve the RSEI of the study area by 0.1 unit, thus improving EEQ. In the future territorial spatial planning and construction of Changsha, the scale of inefficient industrial land should be moderately controlled.

The implementation of the Changsha City master plan (2003–2020) has been completed. Its ecological effects were quantitatively evaluated to support the next stage of territorial spatial planning. Using the RSEI model to evaluate the EEQ due to urban planning implementation is undoubtedly a scientific and feasible method. Based on the above analysis, the following suggestions are made for the future territorial spatial planning and ecological environment protection in Changsha City:

During territorial spatial control, the negative impacts of industrial land expansion should be highlighted. In the planning and construction of Changsha Industrial Park, the industrial land layout should be optimized for organic integration with the urban green space system. Industrial land should be developed in groups, and green ecological corridors or barriers should be planned and built between the groups to avoid the clustering of built-up industrial land. Relying on the natural landscape skeleton of Changsha, the grouping of ecological barriers needs to be planned between the central urban area, the Yuelu area, the Xingma area, and the peripheral groups. Disorderly urban sprawl should be avoided. The city should be developed according to a development path combining centralization and decentralization to form an urban spatial pattern with reasonable structure, developed function, excellent environment, and orderly development.

We should control excessive urban expansion to reduce the impact of urbanization on urban ecosystems, strictly control the total scale of urban construction land to improve intensive and economic land use, fully revitalize the construction land stock to enhance land resource utilization, and prioritize the revitalization of idle land to activate inefficient land. Following territorial spatial planning, future urban development should highlight the ecological protection and improvement of people’s quality of life, improve the level of high-quality urban development, strengthen the planning control and dynamic monitoring of territorial spatial use, pay attention to the linkage effect of regional development and cooperation in the central area, and implement the early warning mechanism for regional ecological environment changes, so as to promote the healthy coexistence of the ecological environment and new urbanization in central China.

## Figures and Tables

**Table 1:** Principal component analysis results of RSEI.

Year	Index	PC1	PC2	PC3	PC4
2003	LSM	0.470	0.479	0.662	−0.329
	NDVI	0.406	−0.517	−0.264	−0.705
	NDBSI	−0.776	−0.071	0.342	−0.524
	LST	−0.100	−0.705	0.610	0.345
	Eigenvalues	0.084	0.048	0.023	0.012
	Percent eigenvalue (%)	87.22	10.05	2.73	0
2008	LSM	0.444	−0.608	0.426	0.502
	NDVI	0.300	−0.056	−0.864	0.399
	NDBSI	−0.530	0.443	0.176	0.767
	LST	−0.280	−0.656	−0.201	0.018
	Eigenvalues	0.310	0.059	0.028	0.004
	Percent eigenvalue (%)	77.31	14.71	6.98	1.00
2014	LSM	0.421	0.183	−0.674	0.380
	NDVI	0.390	0.217	0.714	0.540
	NDBSI	−0.584	−0.256	−0.171	0.751
	LST	−0.373	0.924	−0.082	0.006
	Eigenvalues	0.304	0.055	0.045	0.003
	Percent eigenvalue (%)	76.69	13.51	11.06	0.74
2018	LSM	0.509	0.380	0.503	0.584
	NDVI	0.317	−0.770	−0.287	0.471
	NDBSI	−0.674	0.218	−0.248	0.659
	LST	−0.429	−0.462	0.775	0.005
	Eigenvalues	0.133	0.036	0.026	0.001
	Percent eigenvalue (%)	86.59	9.87	3.54	0

**Table 2:** Average values of the component indicators and RSEI.

Year	Item	NDVI	LSM	NDBSI	LST	RSEI
2003	Mean	0.685	0.503	0.457	0.514	0.532
2008	Mean	0.521	0.531	0.525	0.545	0.515
2014	Mean	0.491	0.529	0.584	0.571	0.500
2018	Mean	0.627	0.496	0.508	0.618	0.523

**Table 3:** Area and percentage changes at each RSEI level in 2003, 2008, 2014, and 2018.

RSEI Level	2003		2008		2014		2018	
	Area (km^2^)	Pct. (%)	Area (km^2^)	Pct. (%)	Area (km^2^)	Pct. (%)	Area (km^2^)	Pct. (%)
Poor (0–0.2)	86.91	2.28	88.815	2.33	226.022	5.93	195.95	5.14
Fair (0.2–0.4)	768.93	20.13	1119.17	29.31	1212.39	31.80	840.59	22.05
Moderate (0.4–0.6)	1599.23	41.88	1195.192	31.30	1081.251	28.36	1378.50	36.16
Good (0.6–0.8)	1104.78	28.93	1139.93	29.85	916.401	24.04	1017.10	26.68
Excellent (0.8–1.0)	259.12	6.78	275.859	7.22	376.157	9.87	380.08	9.97

**Table 4:** Statistical results of RSEI level changes in Changsha during 2003–2018.

Ecological Change Type	Level Change	Level Area (km^2^)	Category Area (km^2^)	Percentage (%)
Degraded	−4	0.28	1027.27	26.94
	−3	8.19		
	−2	115.99		
	−1	902.81		
Unchanged	0	1759.21	1759.21	46.15
Improved	+1	803.84	1025.74	26.91
	+2	190.26		
	+3	29.59		
	+4	2.05		

## Data Availability

The data are contained within the article, and all data sources are mentioned.
